# Lung Auscultation of Hospitalized Patients with SARS-CoV-2 Pneumonia via a Wireless Stethoscope

**DOI:** 10.7150/ijms.54987

**Published:** 2021-01-28

**Authors:** Pengyu Zhang, Bingjian Wang, Yan Liu, Muge Fan, Yong Ji, Hao Xu, Mengdan Xu, Songwen Chen, Qing Li, Zhi Zhang

**Affiliations:** 1 Department of Respiratory and Critical Care Medicine, Shanghai General Hospital, Shanghai Jiaotong University School of medicine (originally named “Shanghai First People's Hospital”), Shanghai, China.; 2 Department of Cardiology, Nanjing Medical University Affiliated Huai'an First People's Hospital, Huai'an, Jiangsu, china; 3 Department of Cardiology , Shanghai General Hospital, Shanghai Jiaotong University School of medicine (originally named “Shanghai First People's Hospital”), Shanghai, China.; 4 Department of Critical Medicine, Shanghai General Hospital, Shanghai Jiaotong University School of medicine (originally named “Shanghai First People's Hospital”), Shanghai, China.

**Keywords:** SARS-CoV-2, auscultation, crackle, stethoscope, pneumonia.

## Abstract

**Objective:** SARS-CoV-2 (originally named COVID-2019) pneumonia is currently prevalent worldwide. The number of cases has increased rapidly but the auscultatory characteristics of affected patients and how to use it to predict who is most likely to survive or die are not available. This study aims to describe the auscultatory characteristics and its clinical relativity of SARS-CoV-2 pneumonia by using a wireless stethoscope.

**Material and methods:** A cross-sectional, observational, single-center case series of 30 consecutive hospitalized patients with confirmed SARS-CoV-2 pneumonia at Leishenshan Hospital in Wuhan, China, were enrolled from March 9 to April 5, 2020. Clinical, laboratory, radiological, treatment data and lung auscultation were collected and analyzed. Lung auscultation was acquired by a wireless electronic stethoscope. Auscultatory characteristics of the moderate, severe, and critically ill patients were compared.

**Results:** Kinds of crackles including fine crackles and wheezing were heard and recorded in these patients. Velcro crackles were heard in most critically ill patients (6/10). Besides, patients with Velcro crackles were all dead (6/6). There was no positive lung auscultatory finding in the moderate group and little positive lung auscultatory findings (4/10) in the severe group.

**Conclusion:** Velcro crackles can be auscultated by this newly designed electronic wireless stethoscope in most critically ill patients infected by SARS-CoV-2 and predicts a poor prognosis. Moderate and severe patients without positive auscultatory findings may have a better prognosis.

## Introduction

Severe acute respiratory syndrome coronavirus 2 (SARS-CoV-2) which was originally named COVID-2019 is an illness that has spread rapidly throughout China and around the world 1. As of Oct 25, 2020, the total number of patients has risen sharply to 43,025,344 globally. Patients infected by SARS-CoV-2 could be mild, moderate, severe, or critically ill 2. Previous studies have already described the epidemiological findings, diagnosis, clinical presentation, and outcomes of patients with SARS-CoV-2 pneumonia 1, 3-5. Usually, the RT-PCR test was the main way to confirm the infection of SARS-CoV-2, CT is also important in diagnosing and determining the severity of the disease 6, 7. However, many critically ill patients in ICU are intubated or under high flow oxygen therapy so it is difficult to do CT in time to monitor the progress of pneumonia. Under such circumstances, doctors should use stethoscopes to detect lung conditions and make decisions 8. However, due to the severe infectious nature of SARS-CoV-2, the patient must be isolated in the ward, so routine auscultation was not possible when doctors were dressed in protective gear 9. For that reason, on one hand, there was in a great need to perform lung auscultation, on another hand 10, there was little data on characterizing the lung auscultation for these patients. However, the relationship between the lung auscultatory characteristics and outcomes of patients with SARS-CoV-2 infection are of paramount importance to treat patients and reduce mortality.

In this study, we investigated the moderate, severe, and critically ill patients with confirmed SARS-CoV-2 pneumonia who were admitted to Wuhan Leishensan hospital. We collected the lung auscultation characteristics among them by a newly designed wireless electronic stethoscope. The data of lung auscultation characteristics of SARS-CoV-2 pneumonia from this study will be of considerable value for predicting who is most likely to survive and who is at risk of becoming critically ill and die.

## Material and Methods

### Study Design and Participants

This single-center observational study was approved by the institutional ethics board of Shanghai General Hospital of Shanghai Jiaotong University and Leishenshan Hospital (No. 202013) which is a designated newly established hospital to treat patients with SARS-CoV-2 pneumonia. Patients confirmed SARS-CoV-2 pneumonia but not mild cases according to WHO interim guidance and Chinese Diagnosis and Treatment Protocol for Novel Coronavirus Pneumonia (CDTPNCP Trial Version 7) were transferred from other hospitals. All consecutive confirmed patients were classified into the three groups (10/group) (moderate, severe, and critically ill), classified according to CDTPNCP Trial Version 7. All patients with a recent documented HRCT evaluation were eligible for the study and enrolled from March 12 to April 5, 2020. They were auscultated by an electronic wireless stethoscope named Stemoscope (Hulu Devices) through an App installed in a smartphone and the results were recorded. Written or oral informed consent was obtained from patients. Exclusion criteria were represented by significant variations in symptoms after HRCT imaging (when possible, a new HRCT was requested); with the preexisted disease which could cause confusing results (i.e. chronic obstructive pulmonary disease, massive hydrothorax, pulmonary tuberculosis, pulmonary hypertension, severe heart failure, and pulmonary heart disease, etc.); pregnant women or children.

### Data Collection

The medical records of all patients with confirmed SARS-CoV-2 infection were reviewed and analyzed by the research team of the Department of Critical Care Unit in Leishenshan Hospital which was taken over by Shanghai General Hospital. Clinical electronic medical records, laboratory findings, HRCT imaging, treatment, and outcomes data were obtained with data collection forms. Data of sex, age, date of disease onset, symptoms, chronic medical histories, vital signs on admission (heart rate, respiratory rate, blood pressure), laboratory values (Complete Blood Count, Basic Metabolic Panel), chest CT scan, and treatment measures during the hospital stay were collected.

**Figure 1:**
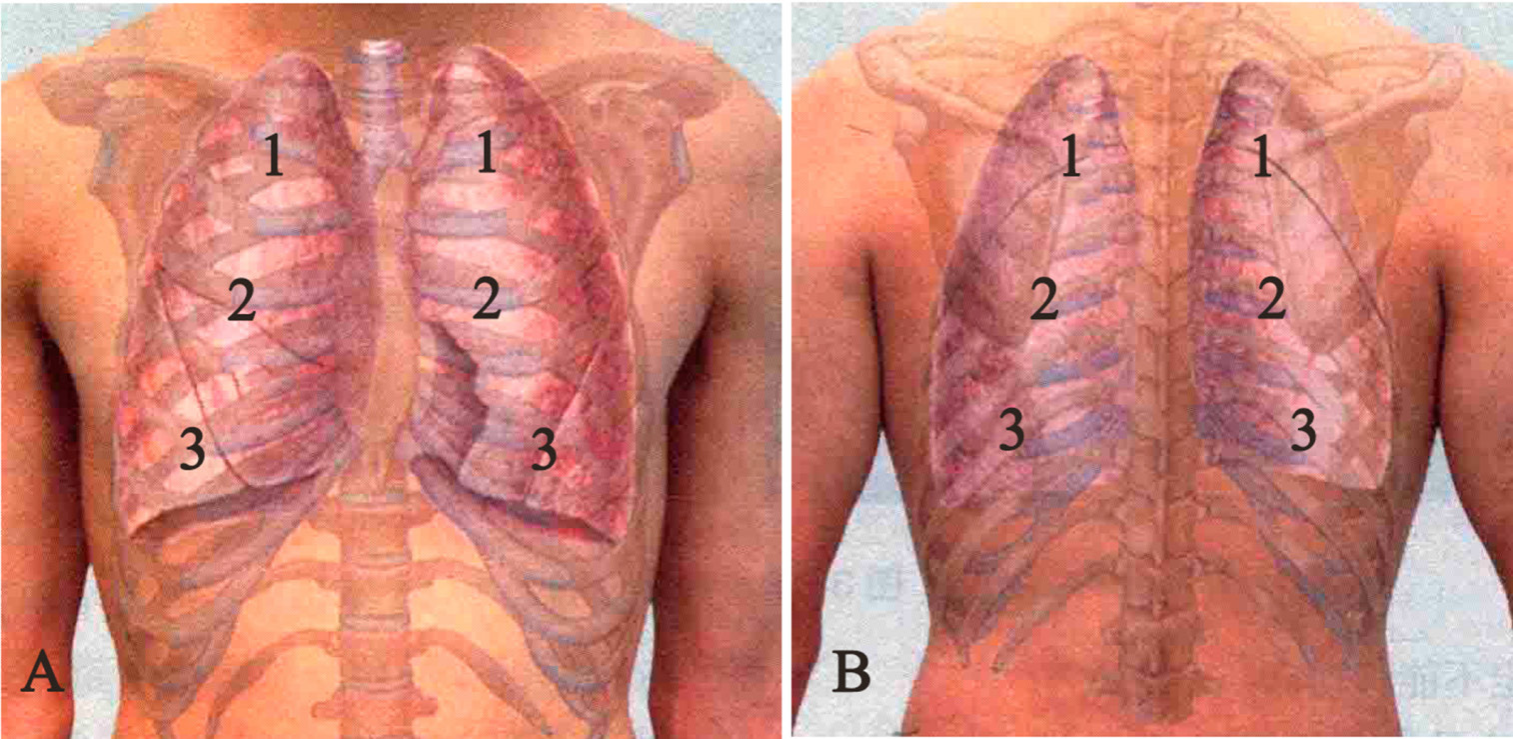
** Respiratory sounds were recorded in 6 pulmonary fields bilaterally.** The total record sites were 12: 2 at the basal field anterior (A) and posterior (B), 2 at the middle field anterior (A) and posterior (B), 2 at the upper field anterior (A) and posterior (B).

Respiratory sounds were recorded in 6 pulmonary fields bilaterally (2 at the basal field anterior and posterior, 2 at the middle field anterior and posterior, 2 at the upper field anterior and posterior (Fig. 1 AB) with a Stemoscope.

**Figure 2:**
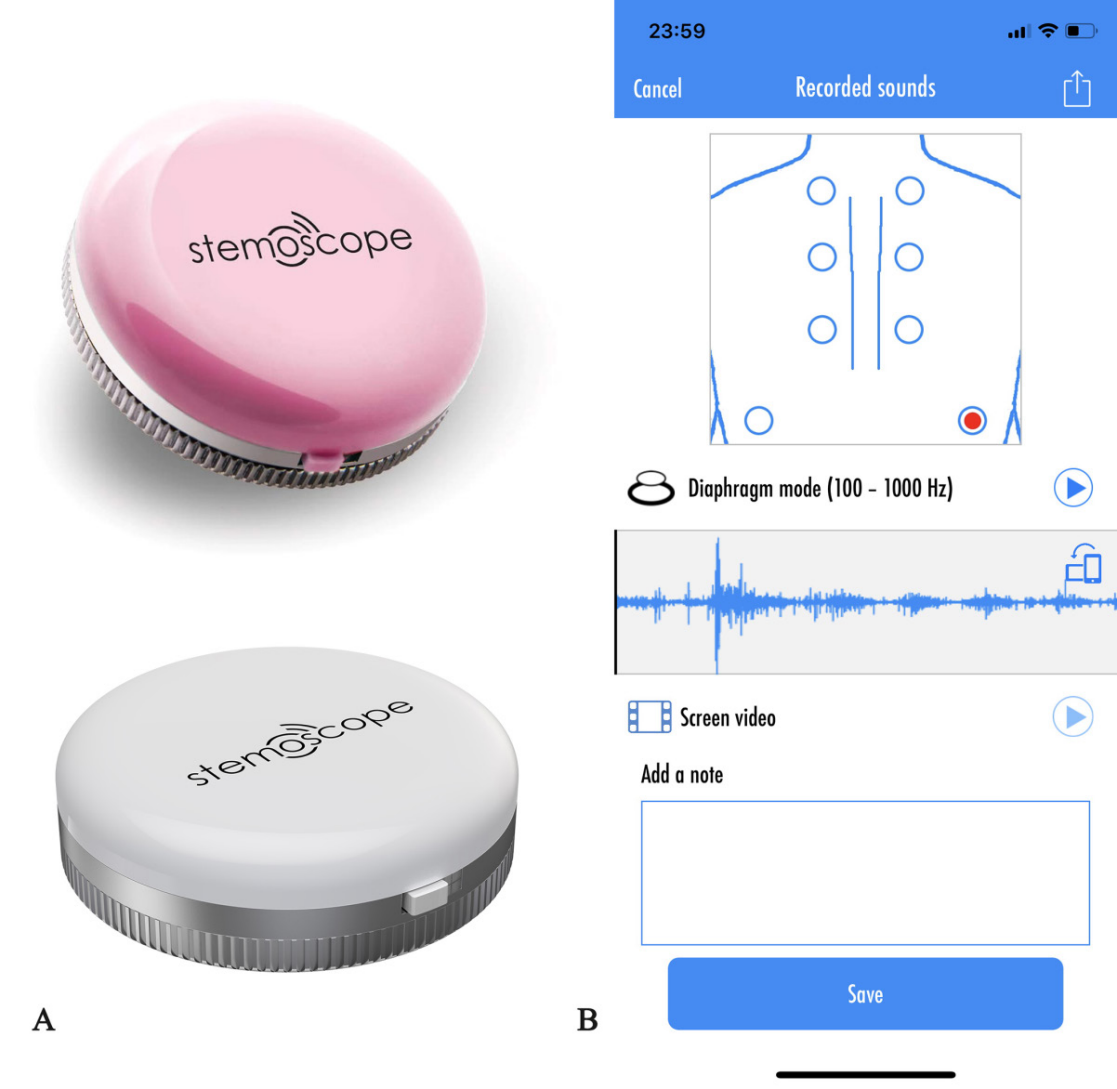
** (A) Stemoscope. (B) Stemoscope App to record the respiratory sounds.** Tap the red dot on the screen to start recording the sounds. Then the investigators can play the recorded sounds. The App provides powerful options to share the recorded sounds such as Twitter, Facebook, and Wechat.

The Stemoscope is made of a Bluetooth hardware device and a software App (Fig. 2 AB). Stemoscope transmits the sounds to the smartphone via Bluetooth. The phone processes and amplifies the sounds and sends them to Bluetooth earphones, Bluetooth hearing aids or wired earphones, in real-time. Tap the red dot on the screen to start recording the sounds. Then the investigators can play the recorded sounds. The App provides powerful options to share the recorded sounds.

**Figure 3:**
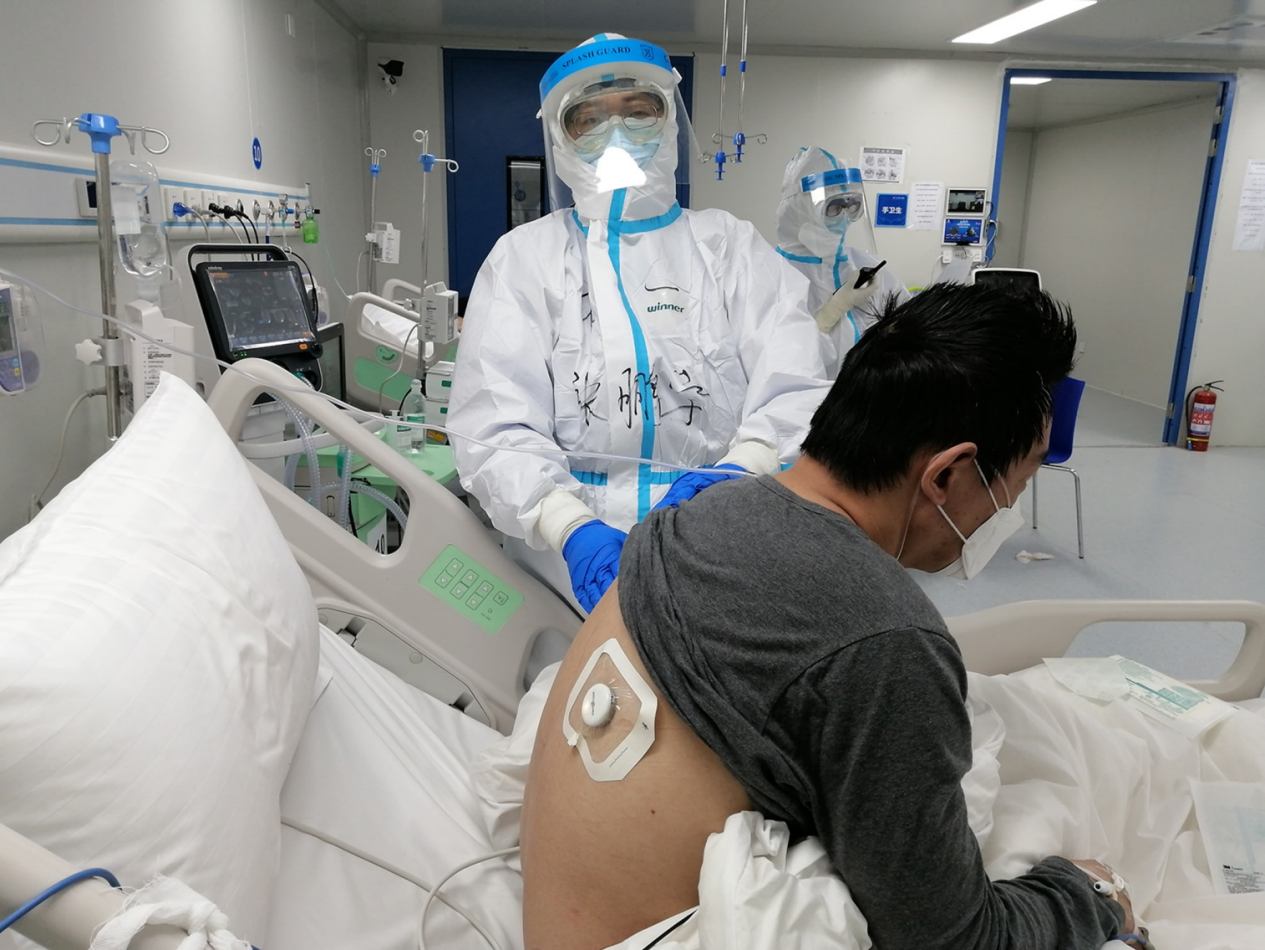
** Respiratory sounds of one SARS-CoV-2 infected patient were recorded by Stemoscope.** The patients were asked to sit upright and his gown was raised to attach the Stemoscope on the skin of his back.

To capture the details of the breath sounds, the extended frequency range mode that covers 20 to 1000 Hz was chosen in the App. The patients were asked to sit upright or elevate the head of bed 45 to 90 degrees for bedridden patients if possible. If patients cannot tolerate sitting, the supine position is allowed for the anterior chest and the side-lying position is used for the posterior chest. The gown of the patients was removed or raised to attach the Stemoscope on the skin (Fig. 3). The subjects followed instructions to breathe deeply for approximately 30s (eight breaths approximately; one breath consists of 2 s-inspiration and 2 s-expiration) to record the lung sounds each site. Then, the video and audio files acquired for each patient were saved, shared, and analyzed by two experts. Respiratory adventitious sounds (type, location, presence on inspiration/expiration, or both) acquired by the microphones were assessed.

All patients underwent scanning with 64-MDCT (GE Healthcare). All imaging data were acquired by scanning with a slice thickness of 0.625-2mm. HRCT exams were performed at full inspiration in the supine position.

All HRCT images were reviewed blindly and independently by two expert radiologists for the assessment of SARS-CoV-2 pneumonia. The distribution of pneumonia was recorded as the focal, multifocal, diffuse pattern. Other pulmonary disease manifestations such as pleural effusions or consolidation also were recorded.

### Statistical analysis

Categorical variables were presented as frequency rates and percentages and were compared by chi-square or Fisher exact test. Continuous variables were presented as Means±SD. Means for continuous variables were compared using independent group t-test or ANOVA when the data were normally distributed; otherwise, the Mann-Whitney test was used. Two-sided *p* < 0.05 was considered statistically significant. All statistical analyses were performed with SPSS software (version 24.0, IBM).

## Results

### Clinical Characteristics

All 30 patients were residents of Wuhan City and transferred from other hospitals with confirmed SARS-CoV-2 pneumonia.

Among the 30 included patients, 10 (8 men, 2 women) were in the moderate group, 10 (6 men, 4 women) were in the severe group and10 (4 men, 6 women) were in the critically ill group. The median age was 61 years (35-92 years). In the critically ill group, the median duration from hospital admission to death was 15 days (5-25 days). Of the 30 patients, only 3 (10%) had no coexisting disease. Hypertension (16 [53%]), diabetes (15 [50%]), cardiovascular disease (8 [27%]) were the most common coexisting diseases (Table 1).

### Vital Signs and Laboratory Parameters in the three groups

Heart rate, high temperature, and oxygen saturation differed among the three groups (Table 1). The initial oxygen saturation was 97±2%, 74±14%, and 55±16% in the moderate, severe, and critically ill group. There were differences in laboratory findings among the three groups (Table 2), including Creatine kinase-MB, Lactate dehydrogenase, and Procalcitonin.

### Main Interventions

Till April 20, 2020, all of 10 patients in the moderate group, 8 patients in the severe group and 1 patient in the critically ill group had been discharged. 2 patients in the severe group and 9 patients in the critically ill group had died. Many patients received antiviral therapy (Arbidol, 20 [66.7%]) and Ribavirin, 14 [46.7%]). Few received Resochin therapy (3 [10%]). Most patients received antibacterial therapy (29 [96.7%]). Many patients in the severe and critically ill groups received glucocorticoid therapy (14 [46.7%]). In the severe group, 1 patient received noninvasive ventilation and 7 received invasive mechanical ventilation. In the critically ill group, 1 patient who was finally discharged received noninvasive ventilation and 9 received invasive mechanical ventilation.

### CT Findings

All patients had CT check after admission except for those already had documented CT images within 24h. The mean interval between admission and CT examination was 0.7 days.

**Figure 4:**
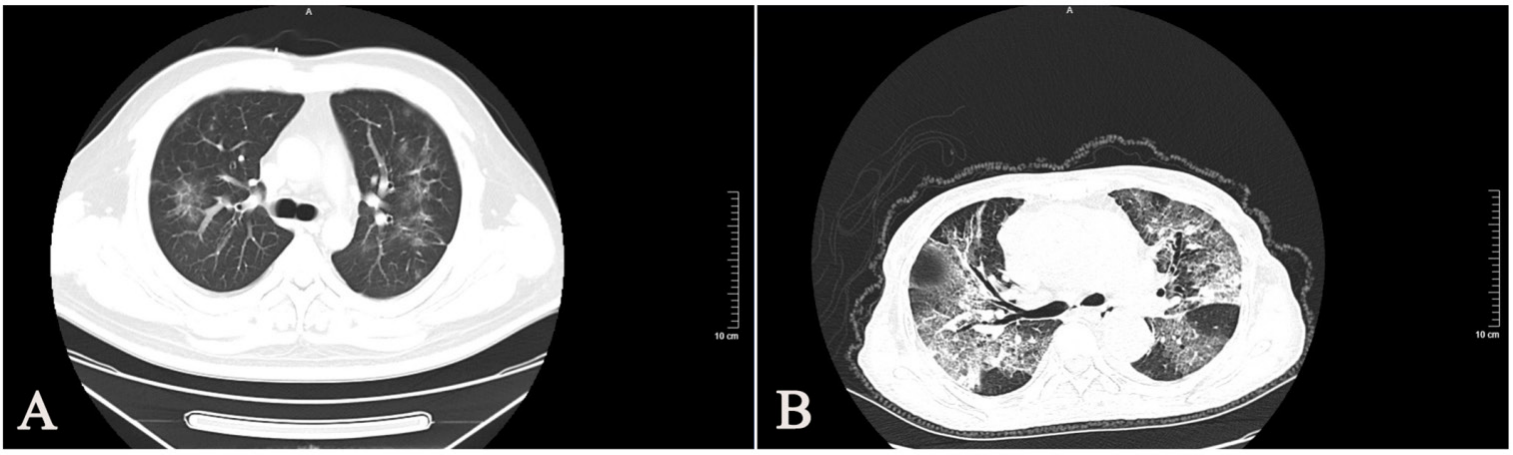
** Chest CT images of SARS-CoV-2 infected patients. (A)** A38-year-old man with confirmed SARS-CoV-2, moderate type. CT images show bilateral diffuse ground-glass opacities. **(B)** A 66-year-old woman with confirmed SARS-CoV-2, critically ill type. CT images show bilateral diffuse ground-glass opacities and reticulation.

All of the 30 enrolled patients showed bilateral involvement of the chest CT scan (Table 1). Most patients with SARS-CoV-2 had typical imaging features, such as GGOs (ground-glass opacities), mixed GGOs, and consolidation (Fig 4). We found that the CT manifestations of severe and critically ill patients were more serious than those of moderate patients.

### Auscultation findings and follow-up

The Stemoscope used in this study was easy and convenient to use. Previous clinical validation showed there was no significant difference between the traditional acoustic stethoscopes and Stemoscope for the lung and heart auscultation.

Crackles (rales) were adventitious sounds that can't be heard in normal individuals while they can exist in pneumonia. 30 patients with SARS-CoV-2 pneumonia were auscultated in this study. In moderate cases, although pneumonia was confirmed by CT, there was no positive finding in lung auscultation (0/10). In server cases that oxygen inhalation was needed to correct hypoxemia, wheezes and fine crackles could be heard (3/10). In critically ill cases that tracheal intubation was needed, all patients have positive auscultation findings. Velcro crackles were the majority (6/10) among which 1 accompanied by wheezes (1/6). Fine crackles also existed (3/10), among which 1 accompanied by wheezes (1/3) (Table 3).

Velcro crackle recorded in one of the patients is listed here: *https://soundcloud.com/zhang-zhi-746982875/Velcro*.

Daily auscultation follow-up of all patients was performed until discharge or death. We found that all moderate patients had no re-occurring positive auscultation findings until discharge. One severe patient with fine crackles died. For one critically ill patient who had only with fine crackles, sputum suction and hormones were used on the top of anti-virus therapy. Then the fine rales became lighter and eventually disappeared and he finally survived. The other six critically ill patients with Velcro rales eventually died.

## Discussion

In this study, SARS-CoV-2 pneumonia patients were successfully auscultated by using a wireless stethoscope. The most important thing was that doctors could do auscultation still being dressed in their personal protective equipment. And for the first time, we found that most of the critically ill patients (60%) had Velcro crackles which maybe had some predictive value.

In our study, the patients who had Velcro crackles all died (6/6), which indicates the poor prognosis. Surprisingly, there was no positive lung auscultatory finding in the moderate group or not much positive lung auscultatory finding (4/10) in the severe group. It seems that patients without positive lung findings may have a better prognosis. However, all the above findings should be interpreted with caution due to the small sample size of this study.

Velcro crackle is a lung sound, defined as a fine crackle that is soft and short in duration, similar to the sound heard when gently separating the strip of velcro attached to the blood pressure cuff (or jogging shoes). It has been proposed as a screening for the diagnosis of interstitial lung disease or pulmonary fibrosis 11, 12. Most patients with SARS-CoV-2 pneumonia had typical GGOs (87%) 13. Interstitial lung disease (ILD) was featured by the occurrence of GGOs 14. And biopsy samples taken from the lungs of SARS-CoV-2 patient showed that interstitial mononuclear inflammatory infiltrates were seen in both lungs 15. All of the above indicated that interstitial invasion was one of the pathophysiological mechanisms of SARS-CoV-2 pneumonia. And it was further confirmed here by Velcro crackles which were characteristic rales of interstitial pneumonia. As a result, detecting Velcro rales in critically ill patients may contribute to understanding the pathophysiological mechanism in SARS-CoV-2 pneumonia.

In our study, there is no significant difference in age, sex, comorbidities among three groups. The top three comorbidities were hypertension, diabetes, and cardiovascular disease which were consistent with previous reports 1, 3. Severe and critically ill groups had a higher body temperature than the moderate group. Other symptoms such as cough, fatigue, dyspnea, expectoration, pharyngalgia don't differ among them. There is no significant difference in systolic, diastolic pressure, respiratory rate when admitted but there is a significant difference in heart rate and oxygen saturation among the three groups. This suggests that high body temperature, heart rate, and low oxygen saturation may be risk factors for poor outcomes.

Compared with CT images, even there were bilateral CT features there was no positive auscultatory finding in moderate and most severe patients. This could be explained by the lung autopsy finding of SARS-CoV-2 that fibrosis and thick secretions were seen in alveoli which didn't produce many fine rales 16.

Most patients in the severe and critically ill groups received two main antiviral therapy, Arbidol, and ribavirin, which was significantly different from the moderate group. Most patients in the severe and critically ill groups also received glucocorticoid therapy while not in the moderate group. Chinese traditional medicine was widely used in all groups without a difference. Invasive mechanical ventilation was required in 16 patients (7/sever group and 9/critically ill group), non-invasive mechanical ventilation was required in 11 patients (7/moderate group, 3/severe group and 1/critically ill group). The low survival rate in critically ill group in this study was partly because the most complicated patients were transferred to Leishenshan hospital. However, this study was not intended to compare the treatment among different groups and it should be also interpreted with caution due to the small sample size of this study.

Stemoscope is a wireless electronic stethoscope which is developed based on our precious validated blood pressure measurement kit 17-19. In this study, we also found some other advantages of Stemoscope. For example, it is difficult to do the CT examination for intubation patients with high oxygen flow. Wireless electronic auscultation can provide timely clinical information for proper intervention. In fact, we successfully relived a severe patient by using glucocorticoid and anti-asthmatic therapy when we auscultated wheezing in her lung. We also detected heart murmurs and confirmed the location of the stomach tube by Stemscope.

According to our findings, moderate patients without rales in their lungs are basically stable. So for infected patients who are isolated at home or have difficulties accessing the hospital could record their sounds and send them to their doctors by a smartphone. Then the doctors could decide whether or not to admit their patients without direct contact. It's maybe an effective way to relieve the medical burden in some areas where SARS-CoV-2 is prevalent. It may also help reduce medical exposure and cross infection.

Our study has some limitations. Firstly, because there were few SARS-CoV-2 patients left in China when the study became, only 30 eligible patients with confirmed SARS-CoV-2 were included in this study. We should need more partners to get further information on SARS-CoV-2 auscultation. Secondly, we don't compare the outcomes of different rales in this study for a long time.

## Conclusion

Velcro crackles can be auscultated in most critically ill patients infected by SARS-CoV-2 and predicted a poor prognosis. Moderate and severe patients without positive auscultatory findings may have a better prognosis. Auscultation is of paramount importance whether in the moderate, severe, or critically ill patients.

## Figures and Tables

**Table 1:** Baseline Characteristics of Patients Infected With SARS-CoV-2

	No. (%)			
	Moderate (n=10)	Severe (n=10)	Critically ill (n=10)	P-value
Age	63±14	63±25	67±10	0.87
**Sex**				
Female	2(20)	4(40)	6(60)	0.19
Male	8(80)	6(60)	4(40)	
**Comorbidities**				
Hypertension	6(60)	6(60)	4(40)	0.59
Diabetes	5(50)	4(40)	6(60)	0.67
Cardiovascular disease	3(30)	3(30)	2(20)	0.84
Cerebrovascular disease	2(20)	3(30)	2(20)	0.83
Malignancy	0	1(10)	2(20)	0.33
**Signs and symptoms**				
Fever	9(90)	10(100)	10(100)	>0.99
**Highest temperature, °C**				
<37.3	2(20)	2(20)	0	0.32
37.3-38.0	5(50)	0	4(40)	0.04
38.1-39.0	3(30)	0	0	0.04
>39.0	0	8(80)	6(60)	<0.01
Fatigue	5(50)	2(20)	7(70)	0.08
Cough	5(50)	3(30)	7(70)	0.20
Dyspnea	3(30)	3(30)	7(70)	0.11
Expectoration	1(10)	0	0	0.36
Pharyngalgia	1(10)	0	0	0.36
Systolic pressure, mm Hg	139±19	138±31	132±20	0.79
Diastolic pressure, mm Hg	82±15	82±7	83±7	0.96
Heart rate,	88±16	104±9	111±21	0.04
Respiratory rate	21±4	21±5	21±6	0.06
Oxygen saturation	97±2	74±14	55±16	<0.01
**Main treatment**				
Arbidol	6(60)	10(100)	4(40)	0.02
Resochin	1(10)	2(20)	0	0.33
Ribavirin	1(10)	7(70)	6(60)	0.02
Glucocorticoid therapy	0	8(80)	6(60)	<0.01
Antibiotic therapy	9(90)	10(100)	10(100)	>0.99
Chinese traditional medicine	9(90)	8(80)	6(60)	0.27
Oxygen inhalation	7(70)	10(100)	10(100)	0.04
NIV	7(70)	3(30)	1(10)	0.02
IMV	0	7(70)	9(90)	<0.01
**CT image**				
Bilateral distribution of patchy shadows or ground-glass opacity, No. (%)	10(100)	10(100)	10(100)	
Survive %	100%	80%	10%	<0.01

Data are Means ± SDs, where N is the total number of patients with available data. P-values comparing the three groups are from chi-square, Fisher's exact test, and ANOVA. NIV = non-invasive ventilation. IMV = invasive mechanical ventilation. P < .05 was considered statistically significant.

**Table 2:** Laboratory Findings of Patients Infected With SARS-CoV-2 on Admission to Hospital

Laboratory Findings	AVERAGE±STDEV.S				
	Normal Range	Moderate (n=10)	Severe (n=10)	Critically ill (n=10)	p-value
White blood cell count, × 10⁹ per L	3.5-9.5	7.8±3.5	6.5±2.6	11.7±5.8	0.12
Neutrophil count, × 10⁹ per L	1.8-6.3	6.0±3.4	5.1±2.6	10.2±5.2	0.07
Lymphocyte count, ×109/L	1.1-3.2	1.1±0.4	0.7±0.2	0.7±0.4	0.11
Monocyte , ×109/L	0.1-0.6	0.7±0.4	0.6±0.2	0.7±0.5	0.84
Haemoglobin, g/L	130-175	102.5±26.7	122.8±23.8	119.4±39.7	0.43
Platelet count, × 10⁹ per L	125-350	255.5±112.6	141.5±59.0	198.5±39.7	0.29
Prothrombin time, s	9-13	11.9±1.5	11.8±0.5	12.3±1.7	0.83
Activated partial thromboplastin time, s	20-40	30.4±8.6	29.9±7.2	28.5±3.4	0.87
D-dimer, mg/L	0-0.55	1.5±1.0	1.6±1.5	4.1±3.1	0.21
Creatine kinase, IU/L	0-171	90.5±80.4	85.0±45.7	25.9±10.7	0.27
Creatine kinase-MB, ng/ml	0-6.36	2.2±1.7	1.9±1.4	9.2±8.0	0.02
Lactate dehydrogenase, U/L	125-243	249.5±92.0	270.0±30.0	415.3±88.7	<0.05
Alanine aminotransferase, U/L	9-50	23.1±10.5	69.0±61.0	34.3±21.0	0.08
Aspartate aminotransferase, U/L	15-40	21.3±12.2	43.7±14.5	34±23.0	0.08
Total bilirubin, mmol/L	2-26	8.6±4.1	12.7±6.7	17.2±4.4	0.01
Blood urea nitrogen, mmol/L	2.8-7.6	9.34±5.5	7.7±3.0	20.4±19.0	0.13
Creatinine, μmol/L	64-104	67.0±11.4	90.8±48.8	69.3±33.7	0.43
Hypersensitive troponin I, ng/mL	0-0.04	0.01±0.0	0.03±0.02	0.1±0.1	0.10
Procalcitonin, ng/mL	0-0.05	0.2±0.2	0.1±0.1	1.0±0.5	<0.01
Potassium, mmol/L	3.5-5.3	4.2±0.9	4.5±0.1	4.5±0.5	0.56
Sodium, mmol/L	137-147	140.6±4.0	138.8±1.0	148.3±16.4	0.63

Data are Means ± SDs, where N is the total number of patients with available data. P-values comparing the three groups are from ANOVA. P < .05 was considered statistically significant.

**Table 3:** Acoustic features of SARS-CoV-2 pneumonia in the three groups

	Moderate	Server	Critically ill	Death
Velcro	0/10	0/10	6/10	6/6
Fine crackle	0/10	3/10	3/10	2/5
Wheeze	0/10	1/10	3/10	1/4
